# Arthroscopic-assisted uniportal spinal surgery for recurrent lumbar schwannoma: a hybrid approach case report

**DOI:** 10.3389/fonc.2026.1884458

**Published:** 2026-09-30

**Authors:** Yubo Zhang, Chen Meng, Yongqiang Li, En Song, Dapeng Li

**Affiliations:** 1 Department of Spine Surgery, Affiliated Hospital of Jiangsu University, Zhenjiang, Jiangsu, China; 2 Department of Sports Medicine, the First Affiliated Hospital of Kunming Medical University, The First Affiliated Hospital of Kunming Medical University, Kunming, Yunnan, China

**Keywords:** AUSS, endoscopic spinal surgery, endoscopic spinal tumor resection, minimally invasive spine surgery, recurrent schwannoma

## Abstract

**Purpose:**

To describe the management of recurrent lumbar schwannomas located within the intertransverse space and psoas compartment using a single-incision hybrid approach that combines open surgery with Arthroscopic-Assisted Uniportal Spinal Surgery (AUSS).

**Methods:**

A 78-year-old woman presented with a 1-month history of low back pain and left-sided radiculopathy. Seventeen years earlier, she had undergone lumbar schwannoma removal. MRI revealed multiple recurrent tumors along the left L4 nerve root, including lesions in the paraspinal and deep intertransverse–psoas compartments. A hybrid surgical strategy was employed: superficial tumors were resected via an open approach, and deeper lesions were removed endoscopically using AUSS through the same incision.

**Results:**

All tumors were successfully excised en bloc. AUSS provided high-definition visualization that facilitated circumferential dissection of a tumor densely adherent to the L4 nerve root. The patient experienced rapid relief of symptoms postoperatively, with complete resolution of radicular pain. Follow-up CT/MRI confirmed full tumor resection and adequate neural decompression. Histopathology confirmed multiple schwannomas.

**Conclusion:**

A combined open–AUSS approach through a single incision offers a feasible and safe option for treating recurrent lumbar schwannomas extending across multiple anatomical compartments. This hybrid strategy minimizes tissue disruption and may represent an effective alternative for managing complex recurrent paraspinal tumors.

## Introduction

Schwannomas are common benign tumors arising from Schwann cells and may involve any segment of the spinal nerve sheath (1). Most lesions present as solitary masses, and primary surgical resection generally enables gross total removal with long-term symptom control. However, a minority of patients experience recurrence, typically related to deep-seated tumor location, complex anatomical interfaces, or incomplete resection during the initial operation (2). Recurrent tumors in the paraspinal or psoas compartments may be accompanied by scar tissue and disrupted tissue planes, making re-exposure more challenging and increasing the risk of neurological injury (3). With advances in minimally invasive spinal techniques, endoscope-assisted tumor resection has been reported in selected schwannoma cases. Arthroscopic-Assisted Uniportal Spinal Surgery (AUSS) combines a limited muscle-splitting corridor with endoscopic magnification and non-coaxial instrument manipulation (4–6). However, its role in recurrent tumors spanning several paraspinal compartments has not been established.

In this report, we describe multiple recurrent lumbar schwannomas involving the paraspinal region, intertransverse space, and psoas compartment, managed through a single posterior incision with a hybrid approach that integrated open surgery and AUSS tailored to the location of each tumor. This report focuses on the rationale and technical feasibility of using direct open visualization for the superficial lesions and AUSS for the deeper lesions. To the best of our knowledge, no directly comparable report has described this specific single-incision combination for recurrent, multinodular lumbar schwannoma spanning the intertransverse and psoas compartments. This case demonstrates the complementary application of open surgery and AUSS based on lesion location and surgical accessibility, providing a potential individualized strategy for the management of complex recurrent lumbar schwannomas with multicompartmental involvement.

## Case presentation

A 78-year-old woman was admitted with a 1-month history of intermittent low back pain radiating to the anterior thigh and anteromedial aspect of the left lower leg. Her symptoms were aggravated by walking or prolonged standing, limiting her walking and standing tolerance to approximately 5 minutes. On admission, the VAS scores were 6/10 for low back pain and 4/10 for radicular pain. Seventeen years earlier, she had undergone resection of a lumbar schwannoma at a local hospital.

Physical examination revealed a soft, mobile subcutaneous mass over the left L4–L5 paraspinal region. Percussion and palpation of the left paraspinal muscles elicited local tenderness and reproduced the radicular symptoms. Neurological examination showed Medical Research Council grade 4/5 strength in the left quadriceps and decreased superficial sensation over the anteromedial aspect of the left lower leg. The left patellar reflex was diminished. The straight-leg-raising test was positive at 60° on the left, and the left femoral nerve-stretch test was also positive. These findings were consistent with left L4 nerve root involvement.

Lumbar CT revealed multiple well-circumscribed, round soft-tissue masses adjacent to the left psoas muscle and paraspinal musculature at the L4–L5 level, without evidence of bony erosion (**Figures 1a, b**). MRI showed lesions that were hypointense on T1-weighted images (**Figures 1e, f**) and hyperintense on T2-weighted and fat-suppressed sequences (**Figures 1c, d, g–j**), with homogeneous enhancement after contrast administration (**Figures 1k–n**). The lesions were located along the expected course of the left L4 nerve root.

**Figure 1:**
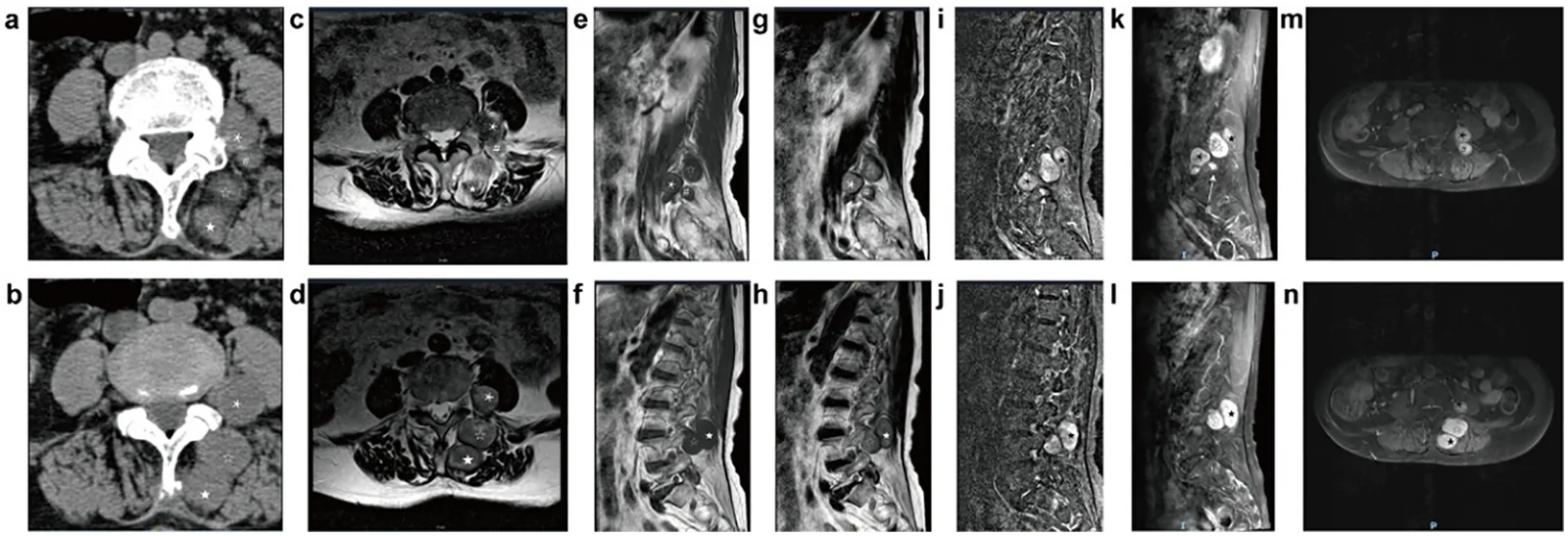
Preoperative imaging evaluation. **(a, b)** Lumbar CT demonstrating multiple well-circumscribed soft-tissue masses on the left side at the L4–L5 level. **(c, d)** Axial MRI showing the distribution of the lesions. **(e, f)** Sagittal T1-weighted images revealing low-signal-intensity lesions. **(g–j)** Sagittal T2-weighted and fat-suppressed sequences showing hyperintense lesions. **(k–n)** Contrast-enhanced MRI demonstrating homogeneous enhancement of all lesions. Tumors are labeled as A1 (*), A2 (#), A3 (☆), and A4 (★) in the corresponding images.

Based on the patient’s previous history of schwannoma resection, the pattern of radicular symptoms, and the characteristic imaging features, recurrent schwannoma was strongly suspected. Considering the different anatomical locations of the recurrent tumors, a hybrid approach was selected. The superficial lesions (A3 and A4) were removed through a limited posterior open exposure, whereas the deep lesions within the intertransverse and psoas compartments (A1 and A2) were addressed using AUSS through the same incision. This approach was designed to facilitate complete resection while reducing unnecessary surgical exposure. Under general anesthesia, the patient was positioned prone, and the L4/5 level was confirmed using C-arm fluoroscopy (**Figures 2a–c**). An approximately 5-cm left paraspinal incision was made, and the paraspinal muscles were split along their fibers. Two superficial tumors (A3 and A4), each measuring approximately 3.5 cm in diameter, were exposed. Both appeared as firm, gray-white, well-encapsulated lesions and were removed en bloc using sharp and blunt dissection (**Figure 2d**).

**Figure 2:**
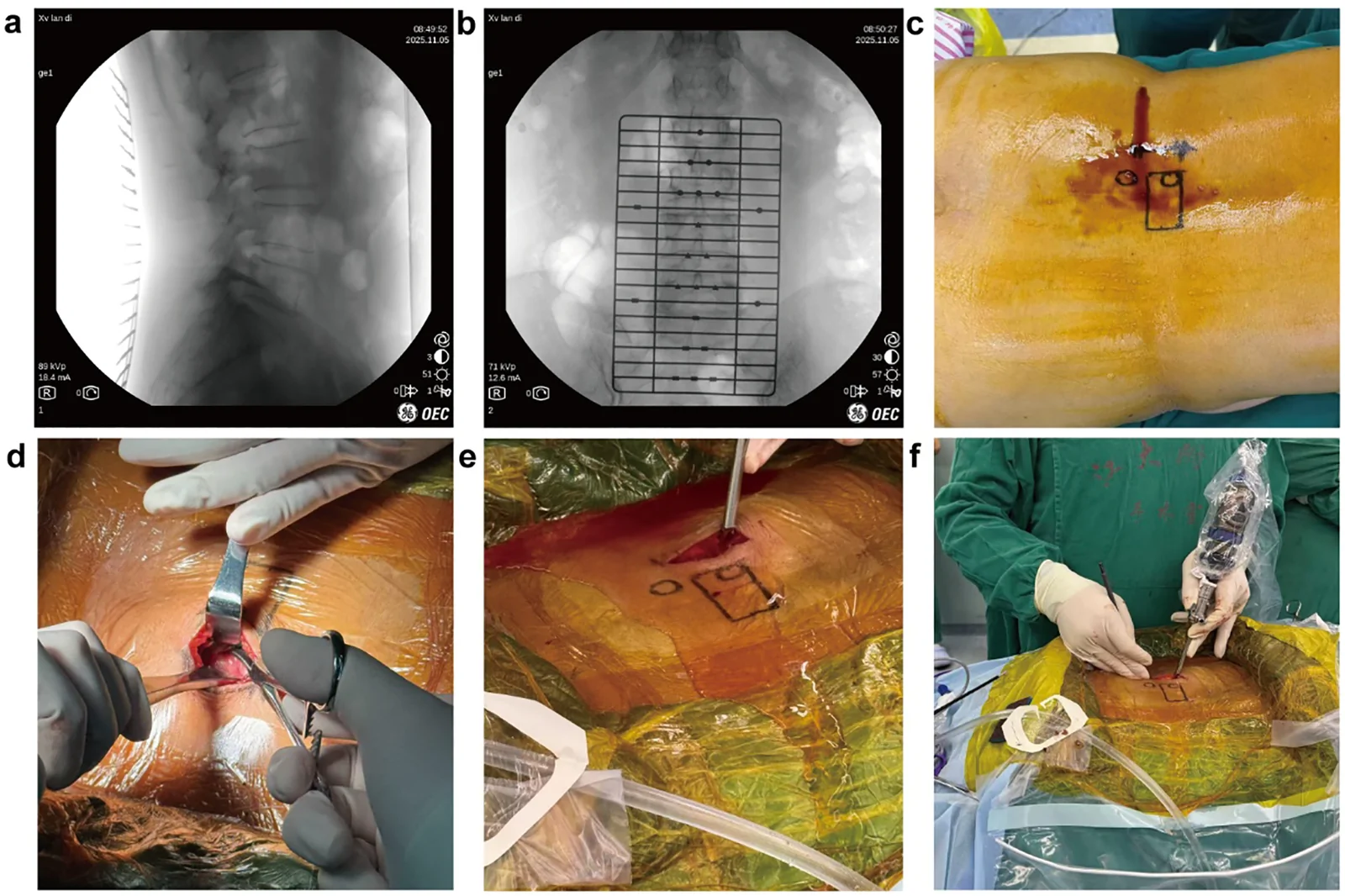
Surgical incision design. **(a–c)** C-arm fluoroscopy used to localize the L4/5 level and determine the skin incision site. **(d)** Exposure of the superficial tumors (A3, A4) through an approximately 5-cm left paraspinal incision. **(e, f)** Introduction of the endoscopic system through the proximal portion of the same incision to establish the working channel for deep tumor resection.

After meticulous hemostasis, the same incision was used to introduce an endoscopic system for resection of the deeper tumors (**Figures 2e, f**). A high-definition arthroscopic system equipped with a 4-mm, 30° rigid arthroscope (Stryker, Kalamazoo, MI, USA) was used. The arthroscope and conventional spinal instruments were introduced independently through the same incision. Continuous gravity-fed irrigation with sterile normal saline was provided by positioning the saline bag 50 cm above the operative field. A bipolar surgical electrode (SDJ-I; Shandong Guanlong Medical Supplies Co., Ltd., Jinan, China) and a disposable radiofrequency plasma surgical electrode (APS-C3-03-S-15035; Suzhou Aikeshuo Technology Co., Ltd., Suzhou, China) were used for soft-tissue ablation and hemostasis.

Endoscopic dissection sequentially exposed the left L5 transverse process, L4 pars interarticularis, L5 superior articular process (**Figures 3a, b**). Two deep-seated tumors were identified within the L4/5 intertransverse space (**Figure 3c**). The distal tumor (A2), located dorsal to the L5 transverse process, showed no attachment to neural structures and was removed first after circumferential dissection (**Figure 3d**). The proximal tumor (A1) extended into the psoas muscle and was firmly adherent to the outer surface of the left L4 nerve root sheath. Its capsule remained intact and was clearly distinguishable from the surrounding neural tissue under endoscopic visualization. The nerve root sheath was not intentionally incised. Under endoscopic magnification, the plane between the intact tumor capsule and the nerve root sheath was progressively developed, primarily by blunt dissection, with limited sharp dissection used only in areas of dense postoperative adhesion. Intraoperative neurophysiological monitoring was not used in this case. The L4 nerve root was identified under direct endoscopic visualization and protected using a UBE nerve root retractor, which was applied primarily as a mechanical barrier rather than for sustained traction. Direct traction, rotation, or coagulation of the nerve root was avoided. Neural tissue was identified primarily by its longitudinal fascicular appearance and continuity with the previously identified L4 nerve root rather than by color alone. When postoperative scarring obscured the tissue plane or tissue identity was uncertain, further manipulation, traction, and coagulation were avoided until the tumor capsule and neural structures could be clearly reidentified. The L4 nerve root was then traced proximally to the lateral entrance of the intervertebral foramen, where no additional tumor was identified. Because preoperative MRI showed no intraforaminal or intraspinal tumor component, further intraforaminal exploration was not undertaken, thereby avoiding unnecessary nerve-root manipulation and additional bony resection. The remaining adhesions were carefully released with a nerve dissector under high-magnification endoscopic visualization, allowing the tumor to be progressively mobilized and removed en bloc (**Figure 3e**). Final endoscopic inspection confirmed adequate decompression of the L4 nerve root, with no gross evidence of traction-related injury (**Figure 3f**). A closed-suction drain was placed, and the wound was closed in layers. Postoperatively, the patient experienced marked relief of symptoms, with VAS scores improving to 1/10 for low back pain and 0/10 for radicular pain. Left quadriceps strength improved from MRC grade 4/5 preoperatively to grade 5/5. However, the decreased superficial sensation over the medial aspect of the left lower leg and the diminished left patellar reflex remained essentially unchanged during the early postoperative period. The drainage volume was approximately 50 mL, and the drain was removed on postoperative day 2. She ambulated on the same day and was discharged uneventfully on postoperative day 5. Follow-up CT and MRI confirmed complete tumor removal and satisfactory decompression of the left L4 nerve root (**Figure 4**). Histopathology revealed multiple schwannomas, with immunohistochemistry positive for S-100 and SOX10 and partially positive for GFAP, while EMA and SSTR2 were negative, consistent with schwannoma (**Figure 5**).

**Figure 3:**
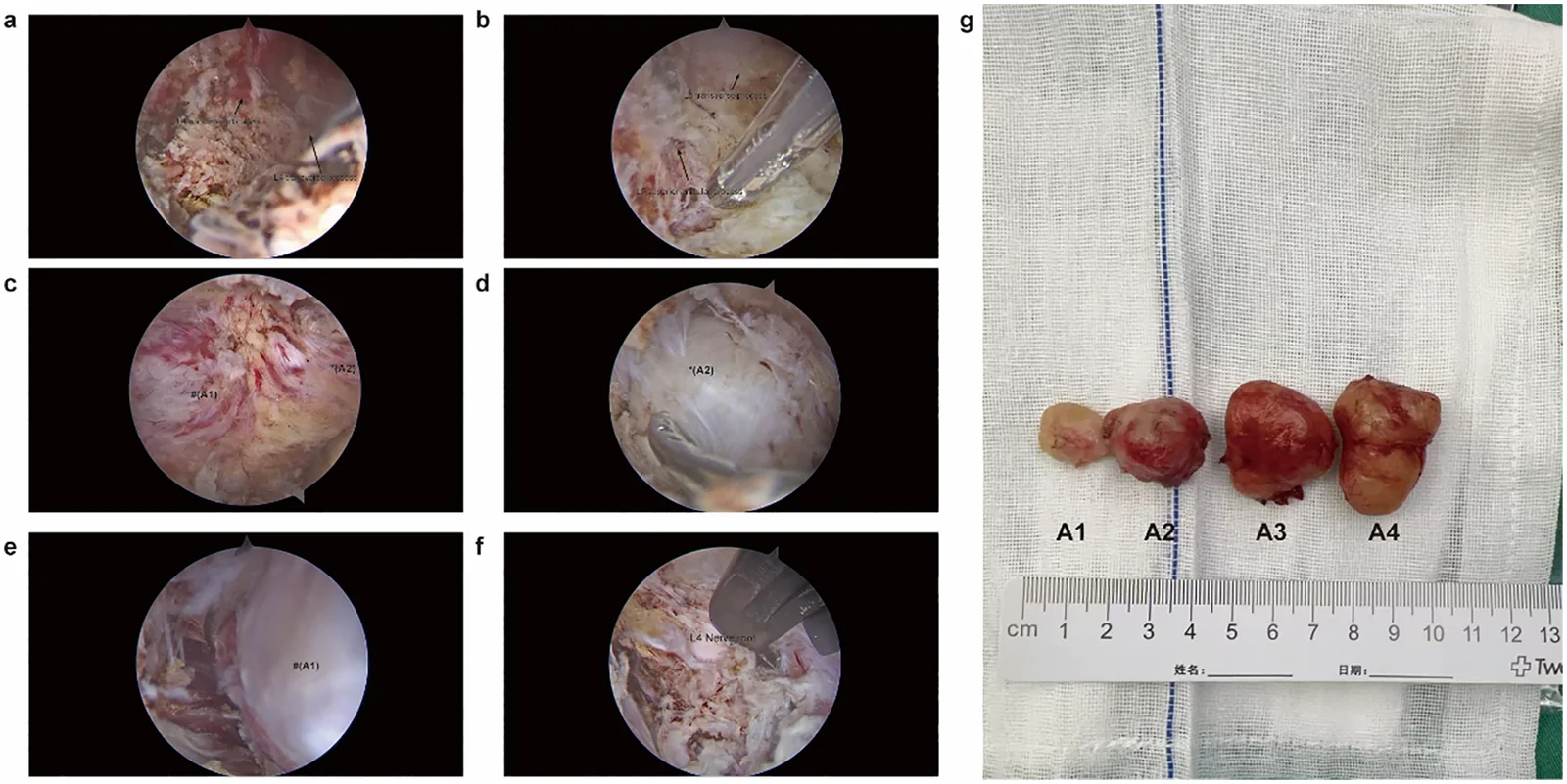
Endoscopic exposure and resection of deep tumors using AUSS. **(a, b)** Endoscopic visualization of the left L4 transverse process, L4 pars interarticularis, L5 superior articular process, and L5 transverse process. **(c)** Identification of two deep tumors located within the L4/5 intertransverse space. **(d)** The distal tumor was removed first after circumferential dissection. **(e)** The proximal tumor was removed en bloc following meticulous endoscopic adhesiolysis. **(f)** Clear visualization of the decompressed left L4 nerve root following removal of both tumors. **(g)** Gross appearance of the completely excised tumors. Tumors are labeled as A1 (*), A2 (#)in the corresponding images.

**Figure 4:**
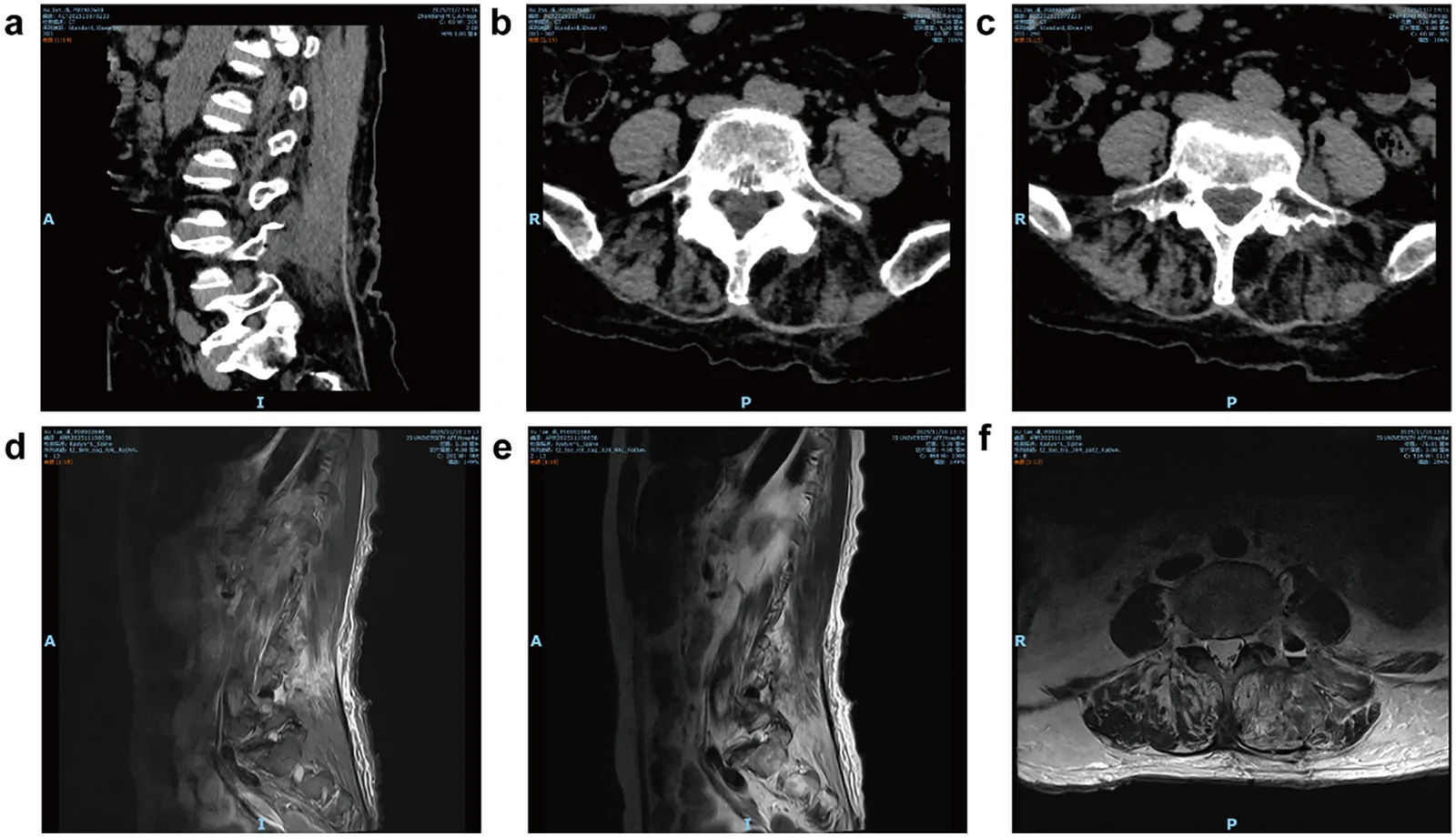
Postoperative imaging review. **(a–c)** Postoperative CT confirming complete removal of both superficial and deep tumors, with no residual lesions. **(d–f)** Postoperative MRI demonstrating complete resection, adequate decompression of the left L4 nerve root, and well-restored paraspinal soft-tissue structures.

**Figure 5:**
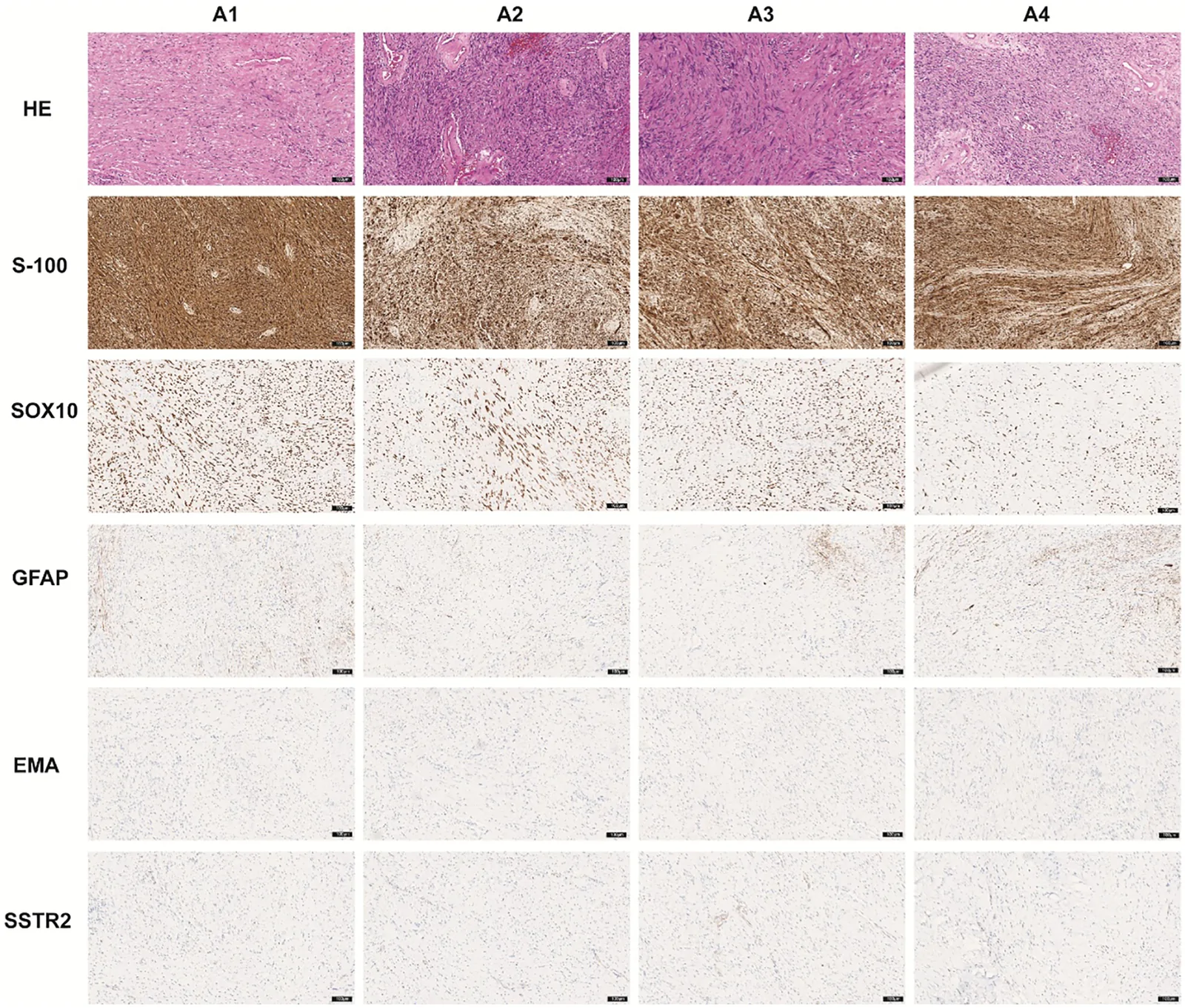
Postoperative histopathology and immunohistochemistry. HE staining showing spindle-shaped tumor cells arranged in intersecting bundles, with characteristic wavy nuclei consistent with schwannoma morphology. Immunohistochemistry demonstrating S-100 (+), SOX10 (+), partial GFAP (+), and negative EMA and SSTR2, supporting the diagnosis of schwannoma.

At the 6-month follow-up, the improvement in low back and radicular pain was maintained, and left quadriceps strength remained at MRC grade 5/5. In contrast to the early postoperative findings, superficial sensation over the medial aspect of the left lower leg had returned to normal, and the left patellar reflex had fully recovered. The patient remained independently ambulatory without any new neurological deficits. Follow-up MRI showed no evidence of residual or recurrent tumor (**Figure 6**).

**Figure 6:**
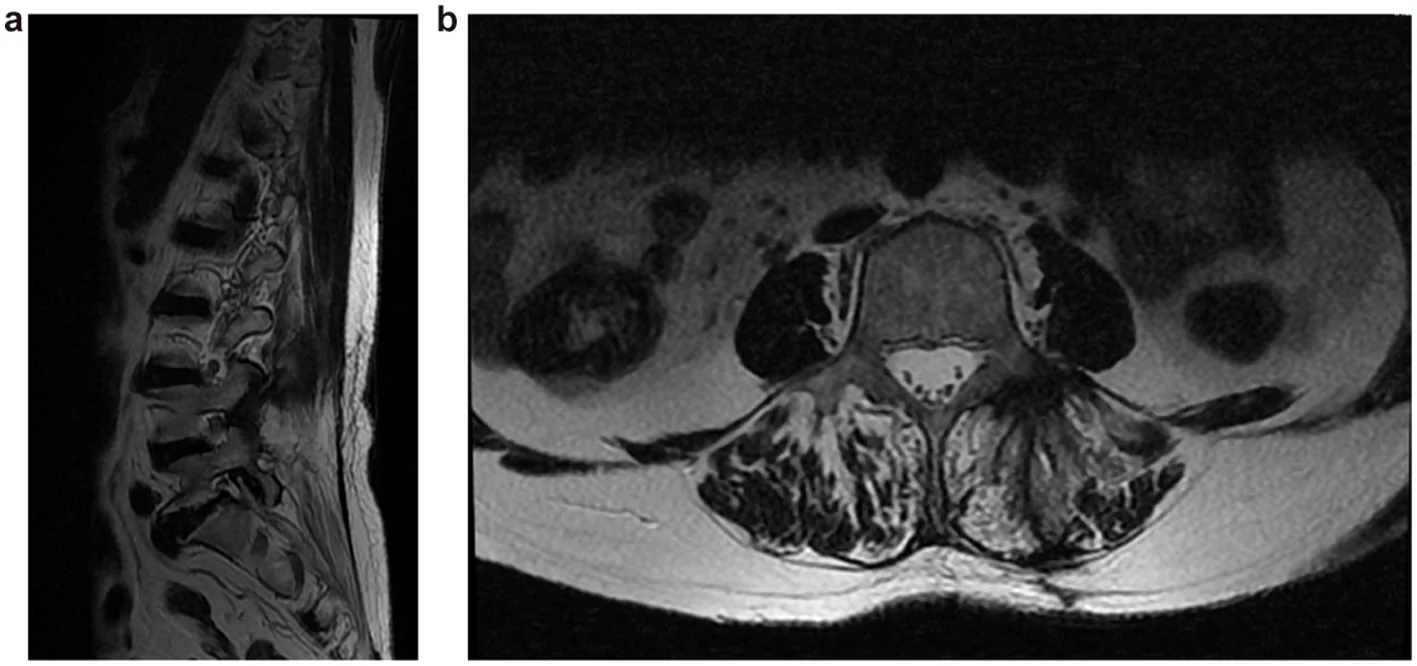
Six-month postoperative follow-up. Lumbar magnetic resonance imaging demonstrated complete tumor resection with no evidence of recurrence.

## Discussion

Schwannomas are slow-growing, benign tumors arising from peripheral nerve sheaths, and recurrence is uncommon after gross total resection at the index operation. Complete microsurgical resection remains the standard treatment for symptomatic spinal schwannomas because the extent of resection influences local tumor control. In a multicenter study of 169 conventional spinal schwannomas, Fehlings et al. reported a recurrence rate of 5.3% and identified larger tumor size and intralesional resection as important risk factors for recurrence (7). Revision surgery for recurrent spinal schwannomas is technically more demanding because postoperative scarring can obliterate normal tissue planes and obscure the tumor–nerve interface (2, 8). Conventional microsurgical revision therefore remains the most versatile approach, providing broad exposure, direct tissue manipulation, reliable hemostasis, and the option to extend the operative field when the anatomy is uncertain. However, when a tumor extends from the posterior paraspinal region into the intertransverse and psoas compartments, adequate exposure of its deep components may require more extensive muscle dissection and prolonged retraction. The surgical objective is therefore not simply to minimize incision length, but to achieve complete resection of each lesion while maintaining adequate control of the tumor–nerve interface and avoiding unnecessary exposure.

Evidence from minimally invasive approaches likewise underscores the importance of tailoring the operative corridor to the location of the lesion. Posterior tubular and far-lateral approaches can provide access to extraforaminal and intertransverse tumors while preserving the facet joint (9). The Wiltse approach has been used for selected schwannomas involving the lumbosacral plexus or psoas muscle (10), whereas direct lateral retroperitoneal and transpsoas approaches offer a shorter and more direct route to tumors centered within the psoas muscle or lumbosacral plexus (11, 12). Landriel et al. treated 23 lumbosacral plexus schwannomas using posterior oblique, transpsoas, or transiliac tubular approaches selected according to tumor level and location, achieving gross total resection in 91.3% of cases (13). These studies provide an important basis for minimally invasive schwannoma surgery. Most reports, however, concern solitary tumors confined to a single anatomical compartment and amenable to treatment through a preplanned, relatively fixed corridor.

Experience with transforaminal full-endoscopic schwannoma resection has progressed from initial feasibility reports to small case series. Wang et al. applied this technique to 12 cases of lumbar dumbbell tumors measuring up to 4 cm (14), Zhou et al. reported complete resection of three lumbar epidural schwannomas (15), and Kravtsov et al. removed a 3.2-cm L5 foraminal schwannoma through an 8-mm incision (16). In the largest series to date, Hu et al. treated 25 foraminal schwannomas using a full-endoscopic technique. Gross total resection was achieved in 24 patients and near-total resection in one; only one patient experienced transient voiding difficulty, and no recurrence or spinal instability was observed during a median follow-up of 22 months (17). Full-endoscopic techniques have also been applied to selected lumbar intradural tumors (18). Collectively, these studies suggest that the transforaminal approach provides a targeted route to tumors centered on or extending continuously through the neural foramen, with the advantages of magnified visualization, limited paraspinal tissue disruption, and reduced facet-joint resection. Nevertheless, the fixed coaxial configuration restricts instrument maneuverability, and a single transforaminal corridor is less suited to recurrent lesions distributed across multiple non-collinear tissue planes (16, 19).

UBE provides another endoscopic option by separating the viewing and working portals, thereby permitting bimanual manipulation with conventional instruments and greater flexibility during dissection and hemostasis. Early reports have described its use for extradural space-occupying lesions (20) and lumbar intradural schwannomas (21). More recently, UBE was used in a series of 11 lower thoracic and lumbar extramedullary tumors, including four schwannomas (22). However, UBE requires the establishment of two portals, and manipulation of large or firm tumors may be technically demanding (19). These limitations may become more pronounced when recurrent tumors consist of multiple nodules distributed across non-collinear tissue planes.

Against this background, AUSS represents a distinct single-incision endoscopic configuration for spinal tumor surgery. Recent reports have described its use for resection of a small intraspinal schwannoma (23) and a far-lateral extraforaminal lumbar chondroma (24). Unlike working-channel full-endoscopic systems, AUSS allows the arthroscope and conventional spinal instruments to be introduced independently through a single incision and manipulated in a non-coaxial manner, combining magnified visualization with the flexibility of familiar spinal instruments.

In the present case, the recurrent tumors were distributed across several anatomical compartments, making it difficult to select a single operative corridor. Conventional open revision, a completely endoscopic procedure, and a hybrid strategy were therefore considered during preoperative planning. Lesions A3 and A4 were relatively large and superficial and were located adjacent to the previous operative field; they could therefore be directly exposed through a limited posterior incision. In contrast, A1 and A2 were located deep within the intertransverse space, with the proximal lesion extending along the L4 nerve root into the psoas compartment. Although a completely open posterior approach could have provided broader exposure, reaching the psoas component might have required more extensive paraspinal muscle dissection and prolonged deep-tissue retraction.

A completely endoscopic procedure offered no clear advantage for the superficial A3 and A4 lesions and would have required multiple tumors in different tissue planes to be addressed through a single fixed corridor. A separate lateral transpsoas approach could have provided direct access to the psoas lesion but would have required an additional surgical corridor without facilitating removal of the superficial lesions. The patient’s advanced age and history of previous surgery further favored limiting unnecessary tissue disruption.

We therefore adopted a hybrid strategy through the same posterior incision: A3 and A4 were removed under direct open visualization, whereas A1 and A2 were resected with AUSS assistance. The open component also preserved the option of extending the exposure if endoscopic visualization, identification of neural structures, or hemostasis proved inadequate. Our literature search identified no directly comparable report describing a single-incision hybrid open–AUSS strategy for multiple recurrent lumbar schwannomas involving the paraspinal, intertransverse, and psoas compartments.

Several limitations should be acknowledged. As a single-case report, this study cannot determine the superiority of the hybrid open-AUSS approach over conventional surgery. The absence of a comparative cohort and the limited follow-up duration also restrict the assessment of long-term tumor control and functional outcomes. Further studies with larger patient populations and longer follow-up are needed to better define its clinical role.

This technique should therefore be considered a complementary option for carefully selected patients with multicompartmental lesions rather than a substitute for conventional microsurgery. Adequate patient selection and intraoperative judgment remain essential, particularly in cases with extensive scarring, unclear tumor–nerve relationships, difficult hemostasis, or insufficient endoscopic visualization. In such situations, extension of the open approach or conversion to conventional microsurgery should be considered to ensure safe and complete tumor removal.

## Conclusion

This case demonstrates the technical feasibility of a compartment-oriented, single-incision hybrid open–AUSS strategy for selected recurrent lumbar schwannomas involving superficial and deep anatomical compartments. Open surgery provided direct control of A3 and A4, whereas AUSS facilitated visualization and dissection of A1 and A2 within the intertransverse and psoas regions. The result should not be interpreted as evidence that the hybrid approach is superior to conventional microsurgical revision, UBE, or full-endoscopic surgery. Larger studies with longer follow-up are required to define its indications, comparative morbidity, neurological safety, and long-term tumor control.

## Data Availability

The original contributions presented in the study are included in the article/**Supplementary Material**. Further inquiries can be directed to the corresponding author.
